# Large Language Models in Neurological Practice: Real-World Study

**DOI:** 10.2196/73212

**Published:** 2025-09-22

**Authors:** Natale Vincenzo Maiorana, Sara Marceglia, Mauro Treddenti, Mattia Tosi, Matteo Guidetti, Maria Francesca Creta, Tommaso Bocci, Serena Oliveri, Filippo Martinelli Boneschi, Alberto Priori

**Affiliations:** 1 Aldo Ravelli Center for Neurotechnology and Experimental Brain Therapeutics, Department of Health Sciences, University of Milan, Via Antonio di Rudinì, 8, Milan, 20142, Italy, 39 02 50323233; 2 Clinical Neurology Unit, Department of Health Sciences, Azienda Socio-Sanitaria Territoriale Santi Paolo e Carlo, University of Milan, Milan, Italy

**Keywords:** neurology, clinical practice, artificial intelligence, large language model, ChatGPT, Gemini

## Abstract

**Background:**

Large language models (LLMs) such as ChatGPT (OpenAI) and Gemini (Google) are increasingly explored for their potential in medical diagnostics, including neurology. Their real-world applicability remains inadequately assessed, particularly in clinical workflows where nuanced decision-making is required.

**Objective:**

This study aims to evaluate the diagnostic accuracy and appropriateness of clinical recommendations provided by not-specifically-trained, freely available ChatGPT and Gemini, compared to neurologists, using real-world clinical cases.

**Methods:**

This study consisted of an experimental evaluation of LLMs’ diagnostic performance presenting real-world neurology cases to ChatGPT and Gemini, comparing their performance with that of clinical neurologists. The study was conducted simulating a first visit using information from anonymized patient records from the Neurology Department of the ASST Santi Paolo e Carlo Hospital, ensuring a real-world clinical context. The study involved a cohort of 28 anonymized patient cases covering a range of neurological conditions and diagnostic complexities representative of daily clinical practice. The primary outcome was diagnostic accuracy of both neurologists and LLMs, defined as concordance with discharge diagnoses. Secondary outcomes included the appropriateness of recommended diagnostic tests, interrater agreement, and the extent of additional prompting required for accurate responses.

**Results:**

Neurologists achieved a diagnostic accuracy of 75%, outperforming ChatGPT (54%) and Gemini (46%). Both LLMs demonstrated limitations in nuanced clinical reasoning and overprescribed diagnostic tests in 17%‐25% of cases. In addition, complex or ambiguous cases required further prompting to refine artificial intelligence–generated responses. Interrater reliability analysis using Fleiss Kappa showed a moderate-to-substantial level of agreement among raters (κ=0.47, SE 0.077; *z*=6.14, *P*<.001), indicating agreement between raters.

**Conclusions:**

While LLMs show potential as supportive tools in neurology, they currently lack the depth required for independent clinical decision-making when using freely available LLMs without previous specific training. The moderate agreement observed among human raters underscores the variability even in expert judgment and highlights the importance of rigorous validation when integrating artificial intelligence tools into clinical workflows. Future research should focus on refining LLM capabilities and developing evaluation methodologies that reflect the complexities of real-world neurological practice, ensuring effective, responsible, and safe use of such promising technologies.

## Introduction

In recent years, large language models (LLMs) and GPT implementations have opened the way for a fluid and natural human-robot interaction [1], with the expectation to further enhance the revolution expected from the massive application of artificial intelligence (AI), also in the health care field [1]. LLMs understand and generate human-like text [2], and are trained on massive datasets, enabling them to perform tasks such as summarization, translation, and conversation [3]. Consumer available LLMs, such as ChatGPT made by OpenAI and Gemini made by Google, enable users to easily interact, enhancing accessibility for both personal and professional purposes.

Physicians might receive rapid support in analyzing clinical data, interpreting results, and formulating hypotheses [4], thus supporting decision-making (diagnosis and treatment) [5] as well as education [6]. For example, LLMs could potentially be used to identify early signs of Alzheimer disease by analyzing patients’ narrative speech or clinical notes, extracting subtle semantic and syntactic markers that might otherwise go unnoticed, or they might assist clinicians in formulating differential diagnoses in complex neurological cases by generating and ranking possible diagnostic hypotheses based on detailed case descriptions.

Ethical considerations and potential risks are, however, around the corner [7]. As a first point, the extensive, but generalist, datasets used for training may limit their performance in specialized domains [8] and may generate an inherent potential for bias [9]. To date, there is a lack of control and transparency on the data used to train consumer-available models [10], thus influencing the accuracy and reliability of the outputs [11]. Another point regards the excessive reliance on these tools that could lead to reduced human oversight and critical thinking, which are of fundamental importance for medical decision-making [12]. In addition, concern resides in the risk that patients with direct access to these models may misinterpret symptoms or engage in self-diagnosis, potentially leading to negative health consequences [13]. Responsible usage, therefore, must be promoted among health care professionals and patients, emphasizing a balanced integration between AI-powered LLMs and human agents [11]. On the other hand, recent studies highlighted the increasing role of LLM-GPTs in the field of neurology, showing promising applications and fast progression across diagnostic and evaluative tasks [14]. For instance, ChatGPT was evaluated on neurology board-style exams and surpassed the human average, showing strength in both lower-order and higher-order reasoning tasks, suggesting potential uses in neurology education and diagnostic support [15].

When evaluating LLMs in the field of neurology, it is crucial to consider that LLMs can be guided using different types of prompts, such as zero-shot prompts, which provide no prior task-specific guides, and few-shot prompts, including examples to help the model understand the request [16]. Structured prompts are highly detailed, specifying the context, the role of the model, and the desired output format, while iterative prompts involve a collaborative refinement process between the user and the model to optimize clarity and effectiveness [16]. The format of input questions or text passed to the model—whether open-ended or multiple-choice—also significantly impacts the accuracy of LLM responses [17]. Open-ended questions allow for a wide range of responses, which can lead to variability in accuracy depending on how well the prompt guides the model. Multiple-choice formats, however, constrain the model to select from predefined options, potentially improving accuracy by limiting the response scope [17]. However, the existing literature on clinical decision-making tools and physician-assistive technologies mostly relies on carefully curated scenarios and inputs designed explicitly for research purposes (eg, educational vignettes or single cases reported in the literature) [18,19]. This, however, differs significantly from the daily routine scenario, in which the doctor in a clinical context (likely even facing the patient) decides to rely on LLM suggestion or assistance and uses unstructured, iterative, human-like interaction styles.

A search of the PubMed database using keywords including “ChatGPT,” “GPT,” and “Gemini” in conjunction with “neurology” yields a rapidly expanding body of literature, particularly from 2019 onward. Within this emerging field, a subset of studies has focused specifically on the diagnostic potential of LLMs or on evaluating their performance using neurology-related questions. Across this literature, a total of 24 studies [14,15,20-41] have explored these applications in greater depth. The studies vary widely in methodology but can be broadly categorized by the type of prompt used and the nature of the clinical material presented to the models. Prompting styles can be grouped into 2 broad, conceptual categories: soft and hard prompting. Soft prompting refers to minimal-input strategies, such as zero-shot or few-shot formats, while hard prompting encompasses more structured, multistep, or multiple-choice inputs, designed to restrict the model’s interpretive latitude and steer its output more precisely. Notably, multiple-choice questions are classified as hard prompts, given their impact on narrowing the interpretive space of the model’s response.

The materials used to test the models’ performance range from real clinical cases to simulated scenarios and standardized exam-style questions. Standardized questions appear in 11 [14,15,24,26-29,31,37,38,41] studies, real cases in 10 [22,23,25,30,32-35,39,40], and simulated cases in 3 [20,21,36]. Hard prompting emerges as the dominant approach, adopted in 18 [14,15,20-25,27,28,30,31,34-39] of the 24 [14,15,20-41] studies—including the majority of those using real or simulated cases (Table 1). By contrast, soft prompting is used in only 5 [26,29,32,33,40] studies, and just 2 [32,33] of these involve real clinical cases.

**Table 1.** Extracted and analyzed studies.

	Authors	Year	Type of prompt	Type of input	Type of cases	LLM^a^	Cases, n
1	Galetta and Meltzer [20]	2023	Hard	Open-ended	Simulated cases	GPT-4	29
2	Chen et al [21]	2023	Hard	Open-ended	Simulated cases	GPT-4	20
3	Patel et al [22]	2024	Hard	Open-ended	Real cases	GPT-3.5	100
4	Du et al [23]	2024	Hard	Open-ended	Real cases	GPT-4 Llama 2	1969
5	Lin et al [24]	2024	Hard	Multiple-choice	Questions	GPT-4o, Claude_3.5 Sonnet, Gemini Advanced	680
6	Wang et al [25]	2024	Hard	Open-ended	Real cases	GPT-3.5, GPT-4	400
7	Tailor et al [26]	2024	Soft	Open-ended	Questions	GPT-3.5, GPT-4, Claude 2, Bing, Bard	21
8	Giannos [27]	2023	Hard	Multiple-choice	Questions	GPT-3.5 Legacy, GPT-3.5 Default, GPT-4	69
9	Chen et al [28]	2023	Hard	Multiple-choice	Questions	GPT-4	560
10	Williams et al [29]	2024	Soft	Open-ended	Questions	GPT-3.5	1
11	Lee et al [30]	2024	Hard	Open-ended	Real cases	GPT-4	46
12	Abbas et al [31]	2024	Hard	Multiple-choice	Questions	GPT-4, GPT-3.5, Claude, Bard	163
13	Shukla et al [32]	2024	Soft	Open-ended	Real cases	GPT-3.5, Microsoft Bing, Google Gemini	10
14	Haemmerli et al [33]	2023	Soft	Open-ended	Real cases	GPT-3.5	10
15	Fonseca et al [14]	2024	Hard	Multiple-choice	Questions	GPT-3.5	188
16	Wang et al [34]	2023	Hard	Open-ended	Real cases	GPT-4, GPT-3.5	174
17	Pedro et al [35]	2024	Hard	Open-ended	Real cases	GPT-3.5	163
18	Nógrádi et al [36]	2024	Hard	Open-ended	Simulated cases	GPT-3.5	200
19	Ros-Arlanzón and Perez-Sempere [37]	2024	Hard	Multiple-choice	Questions	ChatGPT-3.5, ChatGPT-4	80
20	Erdogan [38]	2024	Hard	Multiple-choice	Questions	GPT-4	50
21	Schubert et al [15]	2023	Hard	Multiple-choice	Questions	GPT-3.5, GPT-4	1956
22	Hewitt et al [39]	2024	Hard	Open-ended	Real cases	ChatGPT-4o, Claude-3.5-sonnet, Llama3	30
23	Finelli [40]	2024	Soft	Open-ended	Real cases	GPT-4, GLASS AI	4
24	Altunisik [41]	2024	Soft; Hard	Multiple choice; Open-ended; Binary	Questions	GPT -3.5	216

a LLM: large language model.

Performance outcomes differ significantly depending on both the prompting method and the type of material used. Structured and multiple-choice prompts tend to yield higher accuracy rates. For instance, ChatGPT-4 achieved 100% diagnostic accuracy in a study that relied on structured prompts and multiple-choice formats [31]. In contrast, open-ended approaches applied to real case descriptions have generally led to poorer results. A notable example is a study [40], in which ChatGPT-4 failed to provide any correct diagnoses, while Glass AI produced only one accurate response. Curated prompts and sanitized datasets differ significantly from the high-pressure, overcrowded clinical settings where physicians make rapid decisions under suboptimal conditions. However, considering the everyday scenario, how do these tools perform in the unstructured, fragmented reality of clinical practice? Specifically, when used by fatigued clinicians facing systemic inefficiencies and competing priorities, does their effectiveness decline? Are there associated risks? These questions can be answered only by assessing the diagnostic accuracy and reliability of consumer-available LLMs in real-world applications, using data not specifically prepared, and without a structured interaction workflow.

To fill in this gap, in this study, we focused on Gemini and ChatGPT as the most accessible LLMs and simulated an everyday use. We conducted an experiment comparing them to neurologists on real cases, evaluating their diagnostic accuracy and test recommendations. This simulation does not aim to assess system performance under ideal conditions but rather to examine how these tools integrate into real-world, imperfect settings. Our goal is to uncover critical insights into their usability, reliability, and potential pitfalls when deployed in their intended environments. To this end, we introduce the Unstructured Interaction Paradigm, where LLMs are used in their rawest form to simulate the worst possible misuse scenarios.

## Methods

### Procedure and Case Selection

A total of 56 consecutive patients admitted to the University Hospital Santi Paolo e Carlo - Neurology Department between July 1 and August 31, 2023 were analyzed. Inclusion criteria were that cases should refer to patients who were admitted by a neurologist with a suspected diagnosis, cases should include a discharge letter containing the investigations conducted during the admission and a definitive diagnosis. Patients whose admission was purely therapeutic or observational, without a diagnostic purpose, were excluded. In addition, patients with an already-known diagnosis were excluded.

### Simulation Scenario

**Figure 1.**
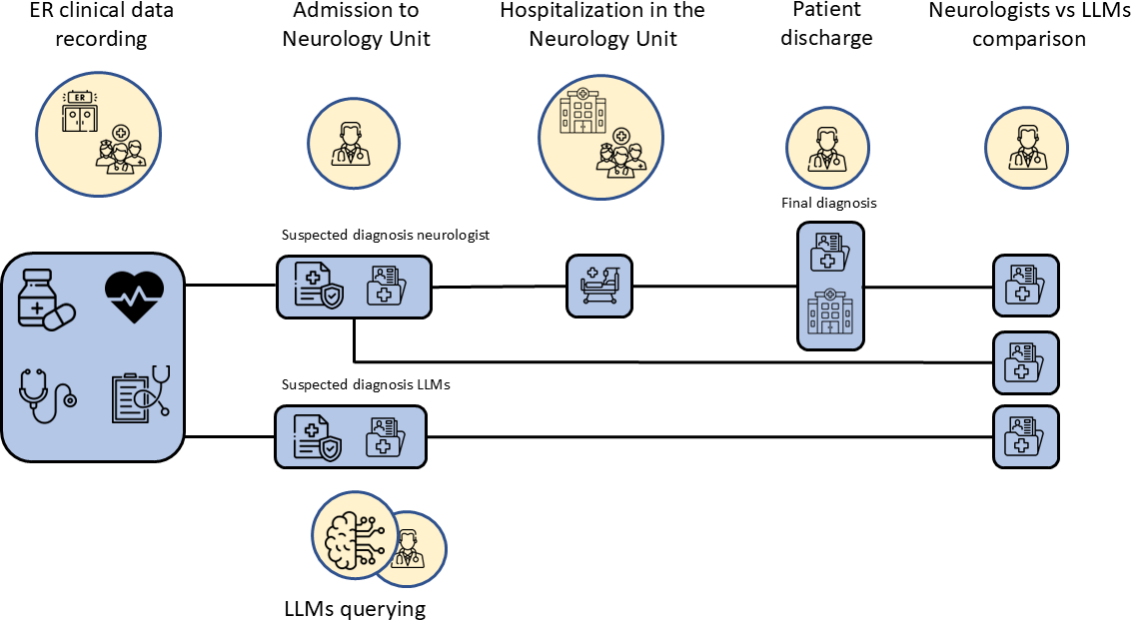
Procedure used to compare diagnosis and clinical tests assessment from neurologists and large language models. ER: emergency room; LLM: large language model.

We retrieved the electronic health records (EHRs) of the selected patients (PDF format) as they were filled in at the time of the patient’s admission. We simulated the process of the initial diagnosis considering the procedure depicted in Figure 1. The patient, coming from the emergency room (ER), is admitted to the Neurology ward. The neurologist in charge of admitting the patient begins the examination by collecting family history, history of present illness, past medical history, and reviewing the diagnostic results performed in the ER. In addition, the neurologist examines lab analysis results when available. At this point, the neurologist formulates a first diagnostic hypothesis that is recorded as the diagnosis at admission.

For each selected patient, the clinical data available to the neurologist at the time of admission were collected. Not all EHRs were structured identically. Some patients had more detailed clinical or family histories or underwent different clinical examinations in the ER.

### Data Presentation: The Unstructured Interaction Paradigm

To simulate the clinical everyday context in which the doctor asks LLM assistance during routine practice, we adopted this Unstructured Interaction Paradigm using freely available models with minimal prompts. Clinical cases were presented to ChatGPT-3.5 (OpenAI) and Gemini, both in their free versions, in September 2024, to assess their reliability in providing neurological diagnoses. These models, based on transformer architectures, have been pretrained on extensive datasets of text and code, developing the ability to understand and process natural language to generate relevant responses. The experimenters accessed the LLMs via their respective websites and interacted with them through the standard dialog box, formulating prompts in the manner of a typical user.

The European TRAIN (Trustworthy and Responsible AI Network) initiative [42] calls for a fundamental shift in how AI is implemented and evaluated in health care. Emphasizing transparency, contextual robustness, and postdeployment monitoring, the TRAIN framework highlights the need to align AI development with the realities of clinical practice and actual user behavior. In line with this, we adopted an unstructured testing paradigm to evaluate LLMs for diagnostic reasoning. Rather than relying on carefully curated prompts or standardized clinical vignettes, our method exposes LLMs to loosely formulated, variably detailed prompts—closer to how both clinicians and lay users are likely to interact with these models in real-world settings. This approach allows us to capture not only performance metrics but also behavioral patterns, biases, and failure points that structured testing may overlook.

While not a substitute for formal validation, unstructured testing complements existing evaluation pipelines by simulating naturalistic interactions. In doing so, it fosters a more trustworthy, transparent, and context-sensitive AI systems testing approach to achieve safe clinical decision-making. The clinical cases were presented by providing the emergency EHR, present and past medical, familial, and social history, which referred patients to the neurology unit for further evaluation. This ensured that both ChatGPT and Gemini received the same information available to neurologists at the time of admission in a raw nonformatted form. EHRs, present and past medical, familiar and social history were directly copied and pasted into the dialog box of the LLMs after being fully anonymized, ensuring compliance with privacy regulations. After the first patient case, subsequent cases were sequentially added in the same session. This methodology aimed to replicate a typical use case in which a physician interacts with the model pragmatically, focusing on obtaining responses rather than ensuring the model’s precise understanding of the input data. Cases were presented by an experimenter unaware of the final diagnosis made by the neurologist to both models under standardized conditions, using the following prompts:

- ChatGPT prompt: *“*Now I will give you a clinical case of a hypothetical patient. You will need to indicate the most likely diagnosis and suggest a series of clinical tests to confirm or rule out the diagnosis. Is that okay?*”*
- Gemini prompt: *“*I’m going to give you some made-up neurological cases. Your job is to tell me what you think is wrong with the patient and what tests you’d order.*”*

The prompts aimed to bypass safeguards preventing harmful diagnostic or treatment suggestions, without specifying cases, formatting, or response structure.

### Outcomes and Data Analysis

The diagnostic accuracy for the neurologists, ChatGPT, and Gemini was evaluated based on their ability to match the patient’s discharge diagnosis. The diagnostic accuracy of the neurologists was evaluated by comparing the suspected diagnosis recorded at the admission to the neurology ward with the discharge diagnosis. To assess the interrater agreement across multiple evaluators, Fleiss Kappa was computed on dichotomous ratings (0=incorrect, 1=correct) provided by 3 independent raters for each of the 28 participants. The evaluation also considered the agreement among neurologists, ChatGPT, and Gemini. In addition, the number of instances in which both LLMs recommended all necessary tests, partially recommended them, or overprescribed unnecessary tests was recorded. Throughout the process, instances where LLMs required additional information or failed to provide a valid response were also documented.

### Ethical Considerations

This study qualifies as a noninterventional observational study and does not constitute a clinical trial. The study has been approved by the Institutional Review Board of the University of Milan with the approval number 123/24, All. 3 CE 10/12/24.

Patients provided consent for the processing of their personal data at the time of hospital admission under protocol ast_daz_502_ed00, in accordance with privacy regulations (D.Lgs. 101/2018, implementing EU GDPR 2016/679). This consent permits research on clinical data. The data have been anonymized prior to processing, ensuring that they cannot be traced back to any specific patient.

## Results

### Case Selection

Clinical case records from 56 patients were analyzed. After applying inclusion and exclusion criteria, we selected 28 patients (mean age 58.2 years; SD 19.1; 16 females) who were eligible for the study (Table 2). Specifically, 19 patients whose admission was solely for therapeutic or observational purposes, lacking a diagnostic intent, were excluded. Furthermore, 9 patients with a pre-existing, confirmed diagnosis prior to admission were also considered not eligible for the study.

**Table 2.** Results comparison between neurologists, ChatGPT, and Gemini.

Case number	Age range (years)	Diagnosis - Neurologist	Diagnosis - Gemini	Diagnosis - ChatGPT	Excessive testing - Gemini	Excessive testing - ChatGPT	Final diagnosis	Diagnostic area	Response issue Gemini	Response issue ChatGPT
1	60‐69	Autoimmune myositis induced by immunotherapy	Autoimmune myositis induced by immunotherapy	Immunotherapy-induced myasthenia gravis	Yes	Yes	Autoimmune myositis induced by immunotherapy	Neuromuscular	N/A^a^	N/A
2	60‐69	Cerebrovascular event	Cerebrovascular event	Cerebrovascular event	No	Yes	Radial nerve mononeuropathy	Neuromuscular	N/A	N/A
3	80‐89	Cerebrovascular event	Cerebrovascular event	Cerebrovascular event	Yes	No	Cerebrovascular event	Vascular	N/A	N/A
4	70‐79	TIA^b^	TIA	TIA	No	No	TIA	Vascular	N/A	N/A
5	70‐79	Cerebrovascular event	Cerebrovascular event	Cerebrovascular event	Yes	No	Focal epileptic seizures in vascular encephalopathy	Epilepsy	N/A	N/A
6	70‐79	Subacute stroke in recent SAH	TIA	Focal seizures with suspected ischemic lesions	Yes	No	Subacute stroke in recent SAH^c^	Vascular	N/A	Tests and diagnosis confirmation
7	70‐79	Acute psychosis	Acute psychosis	Acute psychosis	No	No	Delirium in Mild Cognitive Impairment	Neurodegenerative	N/A	Clarification
8	30‐39	Myasthenia gravis	Fluctuating muscular weakness	Myasthenia gravis	No	No	Myasthenia gravis	Neuromuscular	N/A	N/A
9	60‐69	Delirium in Parkinson disease	Delirium in Parkinson disease	Delirium in Parkinson disease	No	No	Delirium in Parkinson disease	Neurodegenerative	N/A	N/A
10	60‐69	Atypical Parkinsonism	Parkinson disease	Parkinson disease	No	Yes	Atypical Parkinsonism	Movement disorder	N/A	Reprompting
11	70‐79	Ischemic stroke	Ischemic stroke	Ischemic stroke	Yes	Yes	Ischemic stroke	Vascular	N/A	N/A
12	50‐59	TIA	TIA	TIA	No	No	TIA	Vascular	N/A	Reprompting
13	40‐49	Ramsay-Hunt syndrome with VIII c.n. palsy	Ramsay-Hunt syndrome with VIII c.n. palsy	Ramsay-Hunt syndrome with VIII c.n. palsy	No	No	Ramsay-Hunt syndrome with VIII c.n. palsy	Infectious	N/A	N/A
14	80‐89	Ischemic stroke	Intracerebral hematoma following head trauma	Ischemic stroke	No	No	Ischemic stroke	Vascular	N/A	N/A
15	20‐29	SLE^d^	SLE	Optic neuritis	No	No	SLE	Neuroimmunology	N/A	Reprompting
16	70‐79	Delirium	Delirium	Delirium	No	No	Delirium	Neurodegenerative	N/A	N/A
17	30‐39	Autoimmune encephalitis	Autoimmune encephalitis	Autoimmune encephalitis	No	Yes	Acute/subacute schizophrenic episode	Psychiatric	N/A	Reprompting
18	60‐69	Neuromuscular Junction Disorder	Side effects of antiepileptic drugs	Idiopathic generalized epilepsy	Yes	No	Dysarthria with negative work-up	No diagnosis	N/A	Tests and diagnosis confirmation
19	70‐79	Mild Cognitive Impairment	Alzheimer disease	Mild Cognitive Impairment	No	No	Mild cognitive impairment	Neurodegenerative	N/A	N/A
20	40‐49	Stiff person syndrome	Multiple autoimmune diseases: Type 1 diabetes (LADA), mixed connective tissue disease, sagittal sinus thrombosis.	Stiff person syndrome	No	No	Stiff person syndrome	Movement disorder	N/A	N/A
21	40‐49	Ischemic stroke	Ischemic stroke	Vestibular neuritis	No	No	Ischemic stroke	Vascular	N/A	N/A
22	30‐39	CNS^e^ demyelinating disease	CNS lesion	CNS demyelinating disease	No	No	CNS demyelinating disease	Neuroimmunology	N/A	Reprompting
23	80‐89	Ischemic stroke	Ischemic stroke	Ischemic stroke	Yes	No	Ischemic stroke	Vascular	N/A	N/A
24	30‐39	CNS demyelinating disease	Ischemic stroke	Cerebrovascular event	No	No	CNS demyelinating disease	Neuroimmunology	N/A	N/A
25	30‐39	CNS Demyelinating disease	CNS Demyelinating disease	CNS demyelinating disease	No	No	CNS demyelinating disease	Neuroimmunology	N/A	N/A
26	60‐69	Ocular myasthenia gravis	Ocular myasthenia gravis	Ocular myasthenia gravis	No	No	Ocular myasthenia gravis	Neuromuscular	N/A	N/A
27	30‐39	Ischemic stroke	Ischemic stroke	Ischemic stroke	No	No	VZV-related^f^ cerebral vasculitis	Neuroimmunology	N/A	N/A
28	30‐39	Left motor hemisyndrome with loss of consciousness at onset	Complex partial epilepsy with secondary generalization	TIA	No	No	Syncope. Left shoulder periarthritis.	No diagnosis	Reprompting	Reprompting

a N/A: not applicable.

b TIA: transitory ischemic attack.

c SHA: subarachnoid hemorrhage.

d SLE: systemic lupus erythematosus.

e CNS: central nervous system.

f VZV: varicella zoster virus.

### Interaction and Prompting

Both ChatGPT and Gemini understood the prompt without requiring further specifications. ChatGPT responded only with the request of the first clinical case, Gemini responded with an explanation of how the future responses would be organized indicating that the scheme of the response would be composed of 3 sections: diagnostic hypothesis, differential diagnosis, and recommended diagnostic test. After the first response, ChatGPT needed further indication in 9 cases out of 28 to achieve complete responses. In one case (ID=7), the experimenter requested clarification to determine whether the condition was considered primarily psychiatric or neurological. In 6 cases (ID=10, 12, 15, 17, 22, 28), ChatGPT needed to be prompted 2 times to provide both the missed diagnosis and clinical tests. In 2 cases (ID=6, 18), specific clinical tests were solicited to confirm or exclude an unclear diagnosis. Conversely, Gemini generally provided an organized response framework and required explicit prompting to deliver a diagnosis only in one case (ID=28). Neither ChatGPT nor Gemini exhibited hallucinations, defined as nonfactual, nonsensical, or inconsistent responses to a clear prompt [43].

### Diagnostic Accuracy

In relation to the accuracy of the proposed diagnosis, neurologists correctly diagnosed 75% of cases, while ChatGPT achieved an accuracy of 54% and Gemini 46%. The diagnostic accuracy was evaluated across a range of neurological disorders, revealing notable differences in performance. In infectious diseases, all 3 raters—neurologist, ChatGPT, and Gemini—demonstrated equal accuracy, correctly diagnosing 50% of the cases. For movement disorders, both neurologists and ChatGPT correctly diagnosed 75% of the cases, while Gemini’s performance was notably lower, correctly diagnosing only 25%. For neuromuscular disorders, neurologists, ChatGPT, and Gemini all displayed equal accuracy, correctly diagnosing 67% of the cases, leaving 33% of the cases misdiagnosed by each rater. In psychiatric disorders, neurologists and Gemini both achieved a 50% accuracy rate, whereas ChatGPT underperformed, correctly diagnosing only 25% of the cases. In vascular disorders, neurologists and Gemini both had an accuracy of 75%, while ChatGPT performed slightly lower, with a 62% correct diagnosis rate. Overall, neurologists achieved the highest diagnostic accuracy across all pathologies, correctly diagnosing 75% of the cases. ChatGPT followed with an overall accuracy of 54%, and Gemini achieved an accuracy of 46%.

Regarding the diagnostic test indication, both ChatGPT and Gemini recommended the correct set of basic tests in 55% of cases. However, there was agreement between ChatGPT and Gemini in suggesting the correct diagnostic tests in only 32% of cases. In terms of overprescription of diagnostic tests, ChatGPT recommended an excessive number of tests in 17% of cases, while Gemini did so in 25%. In 64% of instances, neither ChatGPT nor Gemini overprescribed unnecessary tests. Gemini alone overprescribed tests in 18% of cases, ChatGPT alone did so in 11%, and both models suggested excessive tests in 7% of cases.

### Interrater Agreement

The observed agreement (P₀) was 0.738, indicating that raters gave the same classification in approximately 74% of the cases. The expected agreement by chance (Pₑ) was 0.504, based on the marginal proportions of the 2 categories (correct classification: P₁=0.583; incorrect classification: P₀=0.405). The resulting Fleiss Kappa was κ=0.472, suggesting a moderate to substantial level of agreement beyond chance. The SE of Kappa was 0.077, yielding a z-value of 6.14 and a *P* value <.001 (*P*=8.19×10⁻¹⁰), indicating that the agreement among raters was statistically significant. In qualitative terms, all 3 (neurologists, ChatGPT, and Gemini) agreed on providing the correct diagnosis in 36% of cases. In 25% of cases, all 3 were incorrect. In 11% of cases, only the neurologists were correct, and both ChatGPT-3.5 and Gemini were incorrect. In another 11% of cases, only ChatGPT was wrong, while both the neurologist and Gemini were accurate, and only Gemini was wrong in 18% of cases. Neither ChatGPT nor Gemini was correct in any of the cases where the neurologist failed to indicate the correct diagnosis.

## Discussion

### Principal Findings

This study aimed to address the critical gap in real-world evidence regarding the use of LLMs in assisting daily clinical practice within a neurological setting. Literature reveals a striking underuse of soft prompts in real-case scenarios, despite their potential to better simulate the complexity of everyday clinical interactions. This gap underscored the need for a more ecologically valid evaluation of LLMs in diagnostic settings.

To bridge this gap, we conducted an experiment designed to test LLM performance under real-world conditions. Our results, obtained from a cohort of patients with diverse neurological conditions, showed a general agreement between neurologists and LLMs, even when operating in nonstructured environments. However, while both neurologists and LLMs achieved comparable accuracy in certain disease categories, such as infectious diseases and neuromuscular disorders, neurologists demonstrated superior performance in movement disorders, psychiatric disorders, and vascular disorders. These conditions often require nuanced clinical judgment, experience in interpreting subtle clinical signs, and the ability to integrate information [44]. These results align with the broader literature on LLMs in specialized medical contexts, where human expertise surpasses LLMs’ performance, especially in areas requiring nuanced clinical judgment, advanced interpretative skills, and integrative diagnostic approaches [45]. Regarding diagnostic agreement, there was substantial variability among the 3 raters. While all 3 agreed on the correct diagnosis in a significant proportion of cases, in no cases was the wrong response given by the neurologists only. Our findings align with studies showing that LLMs’ accuracy in clinical recommendation varies with the severity of the presentation and the complexity of the clinical picture: it performs well when initial symptoms clearly indicate a condition, but less so with more ambiguous cases [46]. LLMs appear accurate on the surface when managing well-known, sharply defined symptoms but often fail to provide appropriate clinical management recommendations [46]. This shortfall likely arises from the lack of deep, discipline-specific knowledge and of a clear understanding of clinical constraints, limiting their ability to navigate patients’ management effectively beyond initial diagnosis [46]. Both models, especially Gemini, showed test overprescription, highlighting LLMs’ inability to apply cost-benefit reasoning in clinical care.

While emphasizing the promise of LLMs in clinical practice, it should be noted that their limitations in handling complex diagnoses and in recommending further diagnostic assessments. When examining which model required the most user intervention, ChatGPT more frequently needed reprompting or clarification. In contrast, Gemini’s responses were more likely to require interpretive integration or to present broader differential possibilities without an outright incorrect answer. This may suggest that Gemini adopts a more cautious diagnostic style, whereas ChatGPT may commit more confidently to specific diagnoses, which can lead to both accurate and inaccurate conclusions depending on the clarity of the prompt. In summary, both LLMs performed well in vascular cases, which represent conditions with high diagnostic standardization. However, Gemini was generally more accurate in rare or complex systemic conditions and required fewer follow-up prompts. ChatGPT, on the other hand, tended to be more confident but also more prone to oversimplification, especially when psychiatric or autoimmune factors were involved. The differences in model behavior highlight complementary strengths and limitations: Gemini appears more conservative and multihypothesis-oriented, while ChatGPT offers more direct answers that may require greater user supervision when clinical ambiguity is high.

LLMs can support clinicians in structured assessment contexts, but they are still limited in their ability to account for the subtle and often ambiguous nature of real-world clinical decision-making [44]. Therefore, the Unstructured Interaction Paradigm may use available LLMs as support, but without additional specific training, this use would not increase diagnostic accuracy. The major risk at present is the overprescription of recommended diagnostic tests that may impact costs associated with the diagnostic pathway.

The ability of LLMs to adopt a human-like interaction pattern can be advantageous in human-machine interaction. However, this same feature also increases the risk of misunderstandings due to natural language, unlike typical clinical decision support systems, which rely on standardized and unambiguous communication protocols. The use of LLMs requires careful management to ensure consistency and prevent misinterpretation of the information they provide. New studies should focus on how models are used by clinicians within a human-in-the-loop framework, testing how experts use LLMs. For example, a study by Goh and colleagues [47] showed that while LLMs alone performed better than clinicians using conventional resources, integrating LLMs with expert oversight could enhance diagnostic accuracy and efficiency, indicating the potential for significant improvements in clinical practice when AI tools are effectively integrated with human expertise.

It is crucial to interpret our findings in light of the limitations of our work. The use of consumer-grade versions of LLMs, accessed via web browsers, implies that the observed performance is specific to the versions available at the time of the study and may evolve with the ongoing development of these technologies. Furthermore, the relatively modest size of our patient sample, particularly regarding the distribution across different neurological diseases, limited our ability to conduct in-depth analyses of diagnostic accuracy for specific conditions. Despite these limitations, our study provides an initial contribution to understanding the potential of LLMs in this clinical context, paving the way for future research with larger datasets and the exploration of more advanced programming interfaces to fully evaluate their capabilities and impact in supporting diagnostic processes in neurology. Another limitation could be that using the discharge diagnosis as the gold standard may introduce bias. However, given the observed level of agreement, we consider it a suitable reference for this analysis.

### Conclusion

In conclusion, despite the limitations, our study explored the application of consumer-grade LLMs, such as Gemini and ChatGPT, as potential diagnostic evaluation tools in the field of neurology. The results obtained suggest a promising capability of these models in analyzing clinical information and generating diagnostic assessments. Future work should focus on refining LLMs’ algorithms to better interpret clinical data and test the reliability of responses using large datasets of real-world clinical cases, creating models that complement human expertise. Also, it will be important to train future neurologists on the correct use of such powerful tools, emphasizing the risks of excessive reliance on the suggested results, and empowering the interaction capability to maximize the accuracy of responses, even when using consumer-grade technologies.

## Abbreviations

AI: artificial intelligence
EHR: electronic health record
ER: emergency room
LLM: large language model
TRAIN: Trustworthy and Responsible AI Network

## References

[1] Zhou L, Pan S, Wang J, Vasilakos AV (2017). Machine learning on big data: opportunities and challenges. Neurocomputing.

[2] Radford A, Wu J, Child R, Luan D, Amodei D, Sutskever I (2019). Language models are unsupervised multitask learners.

[3] Basyal L, Sanghvi M (2023). Text summarization using large language models: a comparative study of MPT-7b-instruct, Falcon-7b-instruct, and OpenAI Chat-GPT models. arXiv.

[4] Zhang S, Song J (2024). A chatbot based question and answer system for the auxiliary diagnosis of chronic diseases based on large language model. Sci Rep.

[5] Goodman RS, Patrinely JR, Osterman T, Wheless L, Johnson DB (2023). On the cusp: considering the impact of artificial intelligence language models in healthcare. Med.

[6] Lucas HC, Upperman JS, Robinson JR (2024). A systematic review of large language models and their implications in medical education. Med Educ.

[7] Liu J, Wang C, Liu S (2023). Utility of ChatGPT in clinical practice. J Med Internet Res.

[8] Rane N, Choudhary S, Rane J (2024). Gemini versus ChatGPT: applications, performance, architecture, capabilities, and implementation. SSRNJ.

[9] Shah SV (2024). Accuracy, consistency, and hallucination of large language models when analyzing unstructured clinical notes in electronic medical records. JAMA Netw Open.

[10] Liesenfeld A, Lopez A, Dingemanse M Opening up ChatGPT: tracking openness, transparency, and accountability in instruction-tuned text generators.

[11] Frosolini A, Gennaro P, Cascino F, Gabriele G (2023). In reference to “Role of Chat GPT in Public Health”, to highlight the AI’s incorrect reference generation. Ann Biomed Eng.

[12] Johnson D, Goodman R, Patrinely J (2023). Assessing the accuracy and reliability of AI-generated medical responses: an evaluation of the Chat-GPT model. Res Sq.

[13] Saenger JA, Hunger J, Boss A, Richter J (2024). Delayed diagnosis of a transient ischemic attack caused by ChatGPT. Wien Klin Wochenschr.

[14] Fonseca Â, Ferreira A, Ribeiro L, Moreira S, Duque C (2024). Embracing the future-is artificial intelligence already better? A comparative study of artificial intelligence performance in diagnostic accuracy and decision-making. Eur J Neurol.

[15] Schubert MC, Wick W, Venkataramani V (2023). Evaluating the performance of large language models on a neurology board-style examination. medRxiv.

[16] Heston TF, Khun C (2023). Prompt engineering in medical education. Insights Med Educ.

[17] Rodrigues L, Dwan Pereira F, Cabral L, Gašević D, Ramalho G, Ferreira Mello R (2024). Assessing the quality of automatic-generated short answers using GPT-4. Comput Educ: Artif Intell.

[18] Madadi Y, Delsoz M, Lao PA (2025). ChatGPT assisting diagnosis of neuro-ophthalmology diseases based on case reports. J Neuroophthalmol.

[19] Kozel G, Gurses ME, Gecici NN (2024). Chat-GPT on brain tumors: an examination of artificial intelligence/machine learning’s ability to provide diagnoses and treatment plans for example neuro-oncology cases. Clin Neurol Neurosurg.

[20] Galetta K, Meltzer E (2023). Does GPT-4 have neurophobia? Localization and diagnostic accuracy of an artificial intelligence-powered chatbot in clinical vignettes. J Neurol Sci.

[21] Chen TC, Kaminski E, Koduri L (2023). Chat GPT as a neuro-score calculator: analysis of a large language model’s performance on various neurological exam grading scales. World Neurosurg.

[22] Patel MA, Villalobos F, Shan K (2024). Generative artificial intelligence versus clinicians: who diagnoses multiple sclerosis faster and with greater accuracy?. Mult Scler Relat Disord.

[23] Du X, Novoa-Laurentiev J, Plasek JM (2024). Enhancing early detection of cognitive decline in the elderly: a comparative study utilizing large language models in clinical notes. EBioMedicine.

[24] Lin SY, Hsu YY, Ju SW, Yeh PC, Hsu WH, Kao CH (2024). Assessing AI efficacy in medical knowledge tests: a study using Taiwan’s internal medicine exam questions from 2020 to 2023. Digit Health.

[25] Wang X, Ye S, Feng J, Feng K, Yang H, Li H (2024). Performance of ChatGPT on prehospital acute ischemic stroke and large vessel occlusion (LVO) stroke screening. Digit Health.

[26] Tailor PD, Dalvin LA, Starr MR (2025). A comparative study of large language models, human experts, and expert-edited large language models to neuro-ophthalmology questions. J Neuroophthalmol.

[27] Giannos P (2023). Evaluating the limits of AI in medical specialisation: ChatGPT’s performance on the UK Neurology Specialty Certificate Examination. BMJ Neurol Open.

[28] Chen TC, Multala E, Kearns P (2023). Assessment of ChatGPT’s performance on neurology written board examination questions. BMJ Neurol Open.

[29] Williams SC, Starup-Hansen J, Funnell JP (2024). Can ChatGPT outperform a neurosurgical trainee? A prospective comparative study. Br J Neurosurg.

[30] Lee JH, Choi E, McDougal R, Lytton WW (2024). GPT-4 performance for neurologic localization. Neur Clin Pract.

[31] Abbas A, Rehman MS, Rehman SS (2024). Comparing the performance of popular large language models on the National Board of Medical Examiners sample questions. Cureus.

[32] Shukla R, Mishra AK, Banerjee N, Verma A (2024). The comparison of ChatGPT 3.5, Microsoft Bing, and Google Gemini for diagnosing cases of neuro-ophthalmology. Cureus.

[33] Haemmerli J, Sveikata L, Nouri A (2023). ChatGPT in glioma adjuvant therapy decision making: ready to assume the role of a doctor in the tumour board?. BMJ Health Care Inform.

[34] Wang C, Liu S, Li A, Liu J (2023). Text dialogue analysis for primary screening of mild cognitive impairment: development and validation study. J Med Internet Res.

[35] Pedro T, Sousa JM, Fonseca L (2025). Exploring the use of ChatGPT in predicting anterior circulation stroke functional outcomes after mechanical thrombectomy: a pilot study. J NeuroIntervent Surg.

[36] Nógrádi B, Polgár TF, Meszlényi V (2024). ChatGPT M.D.: is there any room for generative AI in neurology?. PLoS One.

[37] Ros-Arlanzón P, Perez-Sempere A (2024). Evaluating AI competence in specialized medicine: comparative analysis of ChatGPT and neurologists in a neurology specialist examination in Spain. JMIR Med Educ.

[38] Erdogan M (2024). Evaluation of responses of the large language model GPT to the neurology question of the week. Neurol Sci.

[39] Hewitt KJ, Wiest IC, Carrero ZI (2024). Large language models as a diagnostic support tool in neuropathology. J Pathology Clin Res.

[40] Finelli PF (2024). Neurological diagnosis: artificial intelligence compared with diagnostic generator. Neurologist.

[41] Altunisik E, Firat YE, Cengiz EK, Comruk GB (2024). Artificial intelligence performance in clinical neurology queries: the ChatGPT model. Neurol Res.

[42] van Genderen ME, Kant IMJ, Tacchetti C, Jovinge S (2025). Moving toward implementation of responsible artificial intelligence in health care. JAMA.

[43] Waldo J, Boussard S (2024). GPTs and hallucination: why do large language models hallucinate. Queue.

[44] Hager P, Jungmann F, Holland R (2024). Evaluation and mitigation of the limitations of large language models in clinical decision-making. Nat Med.

[45] Zaboli A, Brigo F, Sibilio S, Mian M, Turcato G (2024). Human intelligence versus Chat-GPT: who performs better in correctly classifying patients in triage?. Am J Emerg Med.

[46] Rao A, Pang M, Kim J (2023). Assessing the utility of ChatGPT throughout the entire clinical workflow: development and usability study. J Med Internet Res.

[47] Goh E, Gallo R, Hom J (2024). Large language model influence on diagnostic reasoning: a randomized clinical trial. JAMA Netw Open.

